# Expression of PD-1/PD-L1 in primary breast tumours and metastatic axillary lymph nodes and its correlation with clinicopathological parameters

**DOI:** 10.1038/s41598-019-50898-3

**Published:** 2019-10-07

**Authors:** Chenxi Yuan, Zhaoyun Liu, Qian Yu, Xinzhao Wang, Mengxue Bian, Zhiyong Yu, Jinming Yu

**Affiliations:** 1 School of Medicine and Life Sciences, University of Jinan-Shandong Academy of Medical Science, Jinan, People’s Republic of China; 2 Department of Radiation Oncology, Shandong First Medical University and Shandong Academy of Medical Sciences, Jinan, People’s Republic of China; 3 Breast Oncology, Shandong Cancer Hospital and Institute, Shandong First Medical University and Shandong Academy of Medical Sciences, Jinan, People’s Republic of China; 4 School of Medicine, Shandong University, Jinan, People’s Republic of China; 5 University of Kentucky College of Medicine, Lexington, Kentucky United States of America

**Keywords:** Oncology, Breast cancer

## Abstract

The aim of this study was to compare the expression of PD-1/PD-L1 in primary breast tumours to that in metastatic axillary lymph nodes and to determine the correlation between the PD-1/PD-L1 status and clinicopathologic characteristics. In total, 47 paired breast tumour and metastatic axillary lymph node samples were collected in this study. Immunohistochemical technology was used to determine the positivity or negativity of PD-1/PD-L1. Other patient information was retrieved from medical records. Significant differences in PD-L1 expression were observed between primary breast tumours and paired axillary lymph nodes. We also observed that the presence of PD-1/PD-L1 positivity in metastatic lymph nodes was significantly associated with poor prognostic features, such as a high Ki-67 index (p = 0.048), a high TNM stage (p = 0.012), a large number of metastatic lymph nodes (p = 0.002), and a high histology grade (p = 0.029). Since heterogeneity exists, it is necessary to determine the PD-L1 status in both the primary tumour and metastatic lymph nodes.

## Introduction

Breast cancer has a high incidence and mortality rate and is estimated to be the most common malignancy in females worldwide^1^. As a complex and highly heterogeneous disease, breast cancer can be classified into different subtypes based on its molecular features^2^. This categorization has great significance in guiding cancer treatment: hormone receptor-positive patients can benefit from endocrine therapy, while targeted therapy has been extensively utilized in human epidermal growth factor receptor-2-overexpressing (HER2+) patients. As triple-negative breast cancer (TNBC) lacks the expression of both hormone and HER2 receptors, chemotherapy remains the first-line systemic therapy. Unfortunately, compared to other breast cancer subtypes, TNBC is more aggressive and has a poorer prognosis. The median overall survival (OS) is approximately 18 months or less^3,4^. Recently, the results of the IMpassion130 trial demonstrated that the combination of nab-paclitaxel with atezolizumab (an anti-PD-L1 antibody) could lead to remarkably prolonged progression-free survival (PFS) in patients with metastatic TNBC^5^, and this clinical benefit was especially significant in the subgroup of PD-L1-positive patients. PD-1 (programmed cell death protein-1)/PD-L1 (programmed cell death ligand-1) is a critical immune modulatory pathway, manifested by its crucial roles in the maintenance of peripheral tolerance^6^ and the process of “immune escape” in cancer patients^7^. PD-1 is expressed by T lymphocytes and several other immune cells, acting as a coregulatory cell surface membrane protein^8^. Several studies have shown that on cancer-specific T lymphocytes, the expression of PD-1 is significantly upregulated^9^. As a ligand of PD-1, PD-L1 is usually expressed by antigen-presenting cells (APCs), such as B lymphocytes, macrophages, and tumour cells^7^. When PD-1 combines with PD-L1, it can attenuate the immune functions of lymphocytes and promote the activities of functioning regulatory T (Treg) cells, leading to inhibition of the immune response^10–12^. Although the exact mechanisms of the PD-1/PD-L1 pathway have not yet been completely revealed, immune checkpoint inhibitors, such as the anti-PD-L1 antibody and the anti-PD-1 antibody, have been developed to block the interaction between PD-1 and PD-L1 and are powerful and capable of producing robust antitumour responses. A number of studies have focused on PD-1/PD-L1 expression in various malignancies, such as lymphoma, melanoma, colorectal cancer and lung cancer. However, few studies have investigated the expression of PD-1 and PD-L1 in breast cancer patients. In view of the significance of the PD-1/PD-L1 axis in the immune environment of cancer^13^, our study aimed to investigate PD-1/PD-L1 expression in both primary breast tumour tissue and ipsilateral axillary lymph node metastases. Moreover, we also elucidated the association between the PD-1/PD-L1 status and clinicopathological characteristics in breast cancer patients.

## Results

### Patient characteristics

Table 1 shows the basic characteristics of the included patients. Among the 47 patients, a large portion of their histology was invasive ductal carcinoma, accounting for 91%. All patients’ histology grades were grade 2 (45%) or grade 3 (55%). Twenty-one patients (45%) had 1–3 metastatic axillary lymph nodes, 9 patients (19%) had 4–9 metastatic lymph nodes, and 17 patients (36%) had more than 10 metastatic lymph nodes. Eleven patients (23%) had vascular nerve invasion. When the molecular phenotypes were taken into account, 10 patients each (21%) were diagnosed with the Luminal A subtype and the HER2 overexpressing subtype, 23 patients (49%) were diagnosed with the Luminal B subtype, and 4 patients (9%) were diagnosed with the TNBC subtype. Other information about the status of estrogen receptor (ER), progesterone receptor (PR), HER2, the Ki-67 index, and epidermal growth factor receptor (EGFR) in both the primary breast tumours and metastatic axillary lymph nodes is illustrated in Table 1.

**Table 1:** Patient Characteristics.

Variable	N	%
**Age**		
≤50	24	51
å 50	23	49
**Primary tumor histology**		
Ductal	43	91
Lobular	4	9
**Histology grade**		
Grade 2	21	45
Grade 3	26	55
**Primary tumor size** cm		
≤2	17	36
å 2 cm, ≤5 cm	24	51
å 5 cm	6	13
**Number of metastatic lymph nodes**		
1–3	21	45
4–9	9	19
≥10	17	36
**LVI**		
Yes	11	23
No	36	77
**TNM stage**		
II	21	45
III	26	55
**Primary tumor ER**		
+	34	72
−	13	28
**Primary tumor PR**		
+	27	57
−	20	43
**Primary tumor HER2**		
+	18	38
−	29	62
**Primary tumor Ki-67**		
<20%	18	38
≥20%	29	62
**Primary tumor EGFR**		
+	13	28
−	34	72
**Metastatic LN ER**		
+	37	79
−	10	21
**Metastatic LN PR**		
+	29	62
− 18 38		
**Metastatic LN HER2**		
+	21	45
−	26	55
**Metastatic LN Ki-67**		
<20%	24	51
≥20%	23	49
**Metastatic LN EGFR**		
+	7	15
−	40	85
**Molecular phenotypes**		
Luminal A	10	21
Luminal B	23	49
HER-2+	10	21
TNBC	4	9

### Distributions of PD-1 and PD-L1 in breast tumours and paired metastatic axillary lymph nodes

In this study, the positive rate of PD-1 expression in primary tumours and matched lymph nodes was 49% (23/47) and 51% (24/47), respectively. As illustrated in Table 2, 19 patients showed positive PD-1 expression in both primary tumour specimens and lymph node specimens, while 19 patients showed negative PD-1 expression in both specimens. The concordance rate of PD-1 expression between breast tumours and paired lymph nodes was 80.8% (38/47), and no significant difference was observed (p = 0.81). With regard to the PD-L1 status, the positive rate in primary tumour tissue and matched lymph node tissue was 29.8% (14/47) and 14.8% (7/47), respectively. Among the patients with positive PD-L1 expression in the primary tumour, 7 also had positive PD-L1 expression in the paired lymph nodes, while 7 were negative. All 33 patients with PD-L1-negative primary tumours also had PD-L1-negative paired lymph nodes. Thus, the concordance rate of PD-L1 expression was 85% (40/47). Statistically significant differences in PD-L1 expression were observed between the primary tumours and paired metastatic lymph nodes (p = 0.016).

**Table 2:** Comparison of PD-1/PD-L1 status between primary tumor and axillary lymph node.

Primary tumor	Paired axillary lymph node			p
	+	−	total	
**PD-1**				
+	19	4	23	
−	5	19	24	0.81
**PD-L1**				
+	7	7	14	
−	0	33	33	**0**.016*

chi-square test was used for comparison. Significant P-value in table is indicated by asterisk (*).

### PD-1/PD-L1 expression in different molecular subtypes

As shown in Tables 3 and 4, 10 patients were diagnosed with the Luminal A subtype, 23 patients were diagnosed with the Luminal B subtype, 10 were diagnosed with the HER2 subtype, and 4 were diagnosed with the TNBC subtype. No significant differences in PD-1 and PD-L1 prevalence were observed between these four subtypes in either the primary tumour or the metastatic lymph nodes.

**Table 3:** PD-1/PD-L1 expression in invasive breast tumor.

Molecular subtype	total number	PD-1 positive (T)	χ2	p	PD-L1 positive (T)	χ2	p
Luminal A	10	4 (40%)	0.98	0.87	2 (20%)	2.02	0.63
Luminal B	23	11 (47.8%)			8 (34.7%)		
HER2	10	6 (60%)			2 (20%)		
TNBC	4	2 (50%)			2 (50%)		

Fisher’ exact test was used for comparison.

No significant difference was observed.

**Table 4:** PD-1/PD-L1 expression in metastatic lymph node.

Molecular subtype	total number	PD-1 positive (LN)	χ2	p	PD-L1 positive (LN)	χ2	p
Luminal A	10	2 (20%)	5.52	0.138	0 (0%)	4.95	0.15
Luminal B	23	14 (60.8%)			4 (17.4%)		
HER-2+	10	5 (50%)			1 (10%)		
TNBC	4	3 (75%)			2 (50%)		

Fisher’ exact test was used for comparison.

No significant difference was observed.

### Correlation analysis of the PD-1/PD-L1 status and clinicopathological parameters in matched primary tumours and metastatic lymph nodes

We carried out a χ2 correlation analysis to determine the association between several clinicopathological parameters and PD-1/PD-L1 expression in primary tumour samples and metastatic lymph node samples. In the primary tumours, no significant associations were found between the positivity of PD-1/PD-L1 and all the included clinicopathological characteristics (Table 5). The presence of PD-L1 positivity in metastatic lymph nodes was significantly associated with poor prognostic features, including a high Ki-67 index (p = 0.048), a high TNM stage (p = 0.012) and a large number of metastatic lymph nodes (p = 0.002). In addition, patient age was associated with PD-L1 expression in metastatic lymph nodes (p = 0.046); in detail, its expression was significantly higher in patients older than 50 years. Regarding the PD-1 status in paired lymph nodes, its presence was correlated with a high histology grade (p = 0.029). No significant correlations were observed between PD-1/PD-L1 expression, the ER status, the PR status, the HER2 status, the EGFR status or lymph-vascular invasion (Table 6).

**Table 5:** Correlationship between PD-1/PD-L1 status in primary tumor (T) and clinicopathological characteristics.

		PD-1(T)	PD-L1(T)
ER(PT)	r	0.17	0.38
	p	0.752	0.726
PR(PT)	r	0.015	0.001
	p	1.000	1.000
HER-2(PT)	r	0.50	0.055
	p	0.556	1.000
EGFR(PT)	r	2.89	2.25
	p	0.111	0.163
Ki-67(PT)	r	1.15	0.78
	p	0.371	0.516
Age	r	0.02	0.52
	p	1.000	0.54
TNM stage	r	0.55	2.05
	p	0.561	0.205
Histology grade	r	2.35	0.75
	p	0.215	0.515
Metastatic LN number	r	0.39	2.18
	p	0.629	0.16
LVI	r	0.89	0.04
	p	0.494	1.000

chi-square test was used for correlation analysis.

**Table 6:** Correlation between PD-1/PD-L1 status in metastatic lymph node (LN) and clinicopathological characteristics.

		PD-1(LN)	PD-L1(LN)
ER(MLN)	r	0.39	0.25
	p	0.724	0.630
PR(MLN)	r	1.21	0.23
	p	0.403	0.766
HER-2(MLN)	r	0.55	0.85
	p	0.561	0.436
EGFR(MLN)	r	1.33	0.002
	p	0.416	1.000
Ki-67(MLN)	r	3.53	4.36
	p	0.082	**0**.048*
Age	r	0.18	4.35
	p	0.773	**0**.046*
TNM stage	r	0.17	6.50
	P	0.772	**0**.012*
Histology grade	r	5.87	0.92
	p	**0**.029*	0.428
Metastatic LN number	r	0.43	8.92
	p	0.524	**0**.002*
LVI	r	1.21	0.12
	p	0.318	1.000

chi-square test was used for correlation analysis. Significant P-value is indicated by asterisk (*).

## Discussion

In 2013, immunotherapy applied in the field of cancer treatment was selected as “the year’s breakthrough” by the editors of Science due to its ability to harness the immune system to battle tumours, marking a turning point in the history of cancer therapy^14^. Whereas PD-1 is recognized as a brake on T cells, drugs that target PD-1 and PD-L1 have demonstrated promising therapeutic effects in some highly immunogenic tumours, such as non-Hodgkin lymphoma, non-small cell lung cancer (NSCLC), and melanoma. In comparison, breast cancer is less immunogenic than other types of cancer. Thus, few clinical trials have demonstrated the clinical benefit of immune checkpoint inhibitors in breast cancer. IMpassion130, a phase III trial of an anti-PD-1 or anti-PD-L1 antibody in metastatic TNBC patients, showed notable benefits of the combination of atezolizumab (a drug targeting PD-L1) and chemotherapy in the PD-L1-positive subgroup^5^. In the PD-L1-positive cohort, patients who received the combinatorial treatment achieved obviously extended PFS and OS. In the atezolizumab-nab-paclitaxel group and the placebo-nab-paclitaxel group, the median PFS was 7.5 months and 5 months, respectively, and the median OS was 25.0 months and 15.5 months, respectively. In addition, the objective response rate was markedly higher (58.9% vs. 42.6%) in the chemotherapy-immunotherapy combination group. Since most benefits were observed in PD-L1-positive patients, it is reasonable to suggest that PD-1/PD-L1 can be a promising biomarker to predict breast cancer responsiveness to PD-1/PD-L1 checkpoint blockade. Therefore, we evaluated the expression status of PD-1 and PD-L1 in breast cancer patients. In our study, PD-1/PD-L1 expression in both primary breast tumours and paired metastatic lymph nodes was investigated, and correlations between PD-1/PD-L1 expression and clinicopathological features were also observed. Since both cancer cells and immune cells can express PD-L1, some studies have investigated the PD-L1 status on both cell types. According to previous research, not all tumours show simultaneous PD-L1 expression on tumour cells and immune cells. For example, Powles T *et al*.^15^ studied PD-L1 positivity in metastatic urothelial bladder cancer patients: the prevalence of positive PD-L1 expression in tumour-infiltrating immunocytes and tumour cells was 27% (55 patients) and 11% (22 patients), respectively. Only 4% of patients had positive PD-L1 expression in both immune cells and tumour cells. The study results of Herbst *et al*.^16^ proved a discrepancy in PD-L1 expression between immune cells and tumour cells in several cancers, including melanoma, gastric cancer, NSCLC, renal cell carcinoma (RCC), head and neck squamous cell carcinoma (HNSCC), and pancreatic cancer. It is still under fierce debate whether positive PD-L1 expression on immune cells or tumour cells is more crucial in predicting the tumour response to anti-PD-L1 blockade. A previous study showed that PD-L1 expression by tumour cells was associated with treatment benefits from anti-PD-L1 therapy. However, the correlation between positive PD-L1 expression by immune cells and the objective response rate did not reach statistical significance in multiple cancers^13^. In contrast, another study showed that PD-L1 expression on immune cells was related to the treatment response to a checkpoint inhibitor, while the association with PD-L1 expression on tumour cells did not reach statistical significance^16^. In view of the above contradictory results, in our study, we analyzed PD-L1 positivity not only in tumour cells but also in immune cells, and did not assess its expression on tumour cells and immune cells individually.

Several studies have reported the expression status of PD-1/PD-L1 in breast cancer patients, but the results did not reach a consensus. According to a retrospective study that enrolled 1091 patients with invasive breast cancer, 27.0% of patients (295/1091) were PD-L1 high, and 73.0% of patients (796/1091) were PD-L1 low (the mean immunoscore for PD-L1 was used as the cut-off)^17^. Another TNBC study showed that 51% of patients (69/106) had any PD-L1 staining, and 26% (35/106) of patients had PD-L1 staining with an H-score of 5 or more; regarding PD-1 expression, 86% of patients had any PD-1 staining, and 50% of patients had PD-1 staining with an H-score of 5 or more^18^. Furthermore, in a cohort of 116 breast cancer patients, the prevalence of PD-1 and PD-L1 expression was 51% and 45%, respectively^7^. Since breast cancer is highly heterogeneous, PD-1/PD-L1 expression may vary among different molecular subtypes. According to a previous study, the expression of PD-1 is the highest (27.4%) in the basal-like subtype, while lowest in the Luminal A subtype (4.7%, p < 0.0001)^19^. Another study showed that the expression rates of PD-L1 and PD-1 in TNBC were 47.8% and 43.5%, respectively, which were significantly higher than the expression prevalence in other subtypes^20^. Kim *et al*. also observed higher PD-L1 expression in the HER2 positive and TNBC subtypes than in the ER+/PR+ subtypes^21^. Similarly, Gatalica *et al*. demonstrated that PD-1 and PD-L1 expression was more common in the TNBC subtype than in the Luminal-like subtypes^7^. In contrast, in a study of 1091 breast cancer patients, the expression rate of PD-L1 was 34.1% in the luminal A subtype, which was higher than that of the other breast cancer subtypes^17^. Unfortunately, perhaps due to our limited sample size, the difference in PD-1 or PD-L1 expression between different subtypes was not statistically significant.

It has been found that PD-1 and PD-L1 expression is frequently discordant between the primary tumour and locoregional disease or distant metastasis in some tumours, such as melanoma^22^ and RCC^23^. To evaluate whether the intrapatient heterogeneity of PD-1/PD-L1 expression exists in breast cancer patients, we compared PD-1/PD-L1 expression between the primary tumour and metastatic axillary lymph nodes. In breast cancer, axillary lymph nodes are often the first and most common sites of metastasis^24^. The involvement of metastatic axillary lymph nodes is an important prognostic factor for disease staging, treatment, and prognosis in breast cancer patients^25,26^. The results of our study indicated that PD-1 expression was concordant between the breast tumour and the axillary lymph nodes, whereas PD-L1 expression was not. Ossama and colleagues also compared the concordance of PD-1/PD-L1 expression between the primary breast tumours and paired metastatic lymph nodes of 41 patients, and complete concordance (34 specimens being negative and 7 being positive in both specimens, respectively) was found in tumour cells but not immune cells^27^. Additionally, another study indicated that PD-L1 expression was more frequent in metastatic axillary lymph nodes and that its expression levels were significantly higher than those in the primary tumours^28^. In our study, we found that PD-L1 expression was more frequent in metastatic axillary lymph nodes than in primary breast tumours. According to our hypothesis, cancer cells must successfully evade immune surveillance to metastasize to regional lymph nodes, and one of the mechanisms involved is the upregulation of the PD-1/PD-L1 signaling pathway. Therefore, cancer cells from axillary lymph nodes are very likely to express a higher level of PD-1/PD-L1. Moreover, continued cancer proliferation within the tumour microenvironment may lead to increased tumour heterogeneity, resulting in a higher likelihood of developing PD-L1 expression discordance between the primary breast tumour and paired lymph nodes^29^. On the other hand, it has been shown that IFN-γ secreted from T-helper 1 cells and other immune cells can upregulate the expression of PD-L1 on basal-like breast cancer cells^30^. In addition, some reports provide evidence for the loss of PTEN as a mechanism in regulating PD-L1 expression in TNBC^31^. Therefore, it may be necessary to test PD-L1 expression not only in the primary site but also in nearby lymph nodes due to intrapatient expression disparity. The successful identification of PD-1/PD-L1 positivity in either primary or metastatic disease would be very helpful in selecting patients who respond well to immunotherapy.

In this study, we also performed a correlation analysis between the status of PD-1/PD-L1 and several critical clinicopathological characteristics in the primary tumours and matched axillary metastatic lymph nodes. Our results showed that the prevalence of PD-1/PD-L1 was associated with several characteristics that also predict a poor prognosis, including a high Ki-67 index, cancer stage, a high histology grade, and a large number of metastatic lymph nodes. Our results were consistent with those from previous studies^19,20^. In addition, some studies have investigated the correlation between PD-1/PD-L1 expression and clinical outcomes. For instance, a meta-analysis showed that PD-L1 positivity was associated with poor disease-free survival (DFS) but not OS^21^. Similarly, the results of another study also indicated that patients with high PD-L1 expression had poor DFS, distant metastasis-free survival (DMFS), and OS compared with those with no PD-L1 expression^32^. Muenst S *et al*. found that the presence of PD-1 was correlated with significantly worse OS (HR = 2.736, p < 0.001). Although we did not observe a significant correlation between PD-1/PD-L1 expression and clinical outcomes, the associations between the presence of PD-1/PD-L1 and poor prognostic parameters indirectly suggest a similar correlation. In further subgroup analyses, the positive expression of PD-1 was correlated with significantly worse OS in the Luminal B HER2-subgroup (p < 0.001), the Luminal B HER2+ subgroup (p < 0.001), and the basal-like subgroup (p < 0.001)^19^. By contrast, Kim *et al*. observed that high PD-L1 expression was related to ER negativity, PR negativity and HER2 positivity^21^. However, we found no relevance between the status of PD-1/PD-L1 and that of ER, PR and HER2. Furthermore, although previous studies have shown relevance between high PD-L1 expression and the presence of EGFR mutations in NSCLC^33^, we did not observe this association in breast cancer.

In our study, a discrepancy in PD-L1 expression in the primary tumours and matched metastatic axillary lymph nodes was observed. Testing the PD-L1 status in both the primary breast tumour and metastatic lymph nodes can help doctors determine patients who may respond well to immunotherapy. However, the value of our study is compromised by its sample size, as only 47 pairs of samples may contribute to disparities between our data and those from other published papers. Therefore, further studies are needed to prospectively evaluate a large number of patients to assess the correlation between PD-1/PD-L1 expression and clinical prognosis.

## Material and Methods

### Patient cohort

Our study received approval from the ethics committe of Shandong Cancer Hospital and Insititute.This study included 47 patients who had invasive breast cancer along with ipsilateral axillary metastatic lymphy nodes treated in our department from June, 2017 to November, 2018. All patients received modified radical surgery. The invasive breast tumor and axillary lymph node metastasis were confirmed by pathology. All the patients received no systemic therapy such as chemotherapy or target therapy before surgery and had no distant metastasis. In total, we examined 94 samples including 47invasive breast cancer samples and 47 paired metastatic lymph node samples. The age of patients and the histopathological features (tumor size, axillary lymph node involvement, lymph-vascular invasion, ER status, PR status, HER2 status, Ki-67 index and p53 status, EGFR status) were extracted from electronic medical records and pathological records.

### Immunohistochemical staining

Formalin-fixed, paraffin-embedded (FFPE) specimens were archived for immunohistochemical analyses. The FFPE samples were cut at a thickness of 4 **μ**m and placed on a coated glass slide. Following this, the tissue section were treated as follows: de-paraffinized with xylene, rehydration,antigen retrieval for PD-L1 and PD-1, washing with 0.1% tween in Dulbecco’s phosphate buffered saline and blocking with 10% goat’s serum for 1 hour at room temperature. Staining of PD-L1 was evaluated in both tumor and stromal cells. PD-1 staining was performed only in mononuclear immune cells. Both stainings were done in the intratumoural region of the tissue sections examined. We assessed the staining intensity together with the percentage of stained cells for improved accuracy. Staining intensity was scored as follows: negative (score = 0), weak (score = 1), moderate (score = 2), and strong (score = 3). For statistical purposes, a dichotomous classification of the stained proteins was adopted: those with zero staining were considered as negative, while those with >1% positive cells of any staining intensity were considered as positive.

### Statistical analysis

SPSS version 24.0 software (IBM Corp., USA) was used in our study for statistic analyses. Comparisons of PD-1/PD-L1 expression between primary tumors and metastatic axillary lymph nodes, as well as the relevance between PD-1/PD-L1 presence and several clinicopathological characteristics were evaluated by chi-square test and, where necessary, Fisher’s exact test. Significance was considered when p < 0.05.

### Ethical approval

All procedures performed in studies involving human participants were in accordance with the ethical standards of Ethic Committee of Shandong Cancer Hospital and Institute and with the Declaration of Helsinki prosecuted in 1964.

### Informed consent

Informed consent was acquired from all 47 participants included in the study.
